# Delivery of RNAi Therapeutics to the Airways—From Bench to Bedside

**DOI:** 10.3390/molecules21091249

**Published:** 2016-09-20

**Authors:** Yingshan Qiu, Jenny K. W. Lam, Susan W. S. Leung, Wanling Liang

**Affiliations:** Department of Pharmacology and Pharmacy, Li Ka Shing Faculty of Medicine, The University of Hong Kong, 21 Sassoon Road, Pokfulam, Hong Kong, China; qiuysh@hku.hk (Y.Q.); jkwlam@hku.hk (J.K.W.L.); swsleung@hku.hk (S.W.S.L.)

**Keywords:** RNA interference, short interfering RNA (siRNA), short hairpin RNA (shRNA), microRNA (miRNA), pulmonary delivery, respiratory diseases, inhalation

## Abstract

RNA interference (RNAi) is a potent and specific post-transcriptional gene silencing process. Since its discovery, tremendous efforts have been made to translate RNAi technology into therapeutic applications for the treatment of different human diseases including respiratory diseases, by manipulating the expression of disease-associated gene(s). Similar to other nucleic acid-based therapeutics, the major hurdle of RNAi therapy is delivery. Pulmonary delivery is a promising approach of delivering RNAi therapeutics directly to the airways for treating local conditions and minimizing systemic side effects. It is a non-invasive route of administration that is generally well accepted by patients. However, pulmonary drug delivery is a challenge as the lungs pose a series of anatomical, physiological and immunological barriers to drug delivery. Understanding these barriers is essential for the development an effective RNA delivery system. In this review, the different barriers to pulmonary drug delivery are introduced. The potential of RNAi molecules as new class of therapeutics, and the latest preclinical and clinical studies of using RNAi therapeutics in different respiratory conditions are discussed in details. We hope this review can provide some useful insights for moving inhaled RNAi therapeutics from bench to bedside.

## 1. Introduction

**Figure 1:**
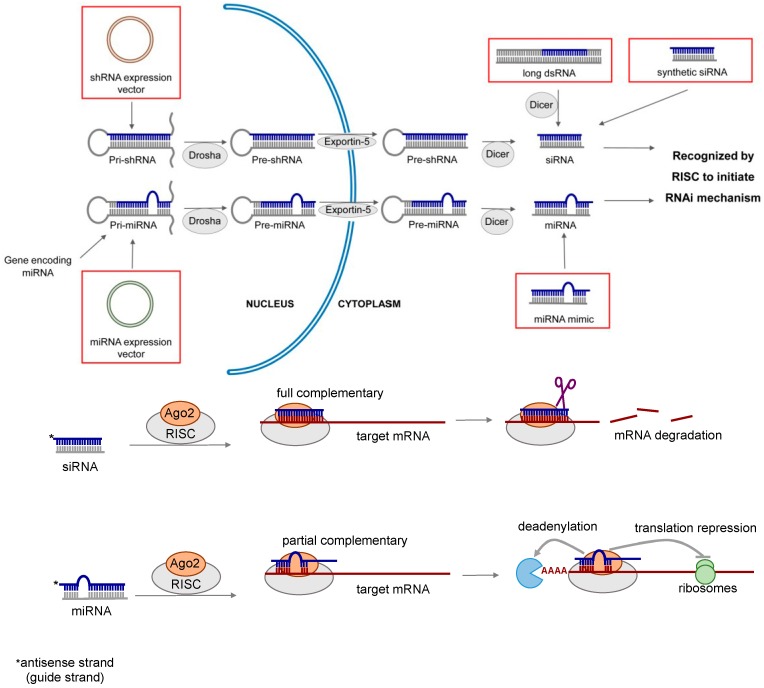
Mechanism of RNA interference (RNAi) induced gene silencing. Short interfering RNA (siRNA) can be produced inside the cells from double stranded RNA (dsRNA) or expressed from short hairpin RNA (shRNA) vector, or chemically synthesized in the laboratory. Its gene silencing effect is dependent upon fully complementary binding of the target messenger RNA (mRNA). MicroRNA (miRNA) is a naturally occurring molecule. It can also be expressed from miRNA vector or chemically synthesized (miRNA mimic). miRNA mediates gene silencing via the partial complementary binding of the target mRNAs.

Lung diseases are among the leading causes of death worldwide. Current treatment of lung diseases such as lung cancer [1], respiratory infections [2,3], inflammatory diseases [4,5] and pulmonary fibrosis [6] have limited efficacy. Almost two decades ago, double-stranded RNAs (dsRNAs) were discovered to play an important role in regulating gene functions by a sequence-specific post-transcriptional gene silencing mechanism called RNA interference (RNAi) [7]. The therapeutic potential of RNAi was soon realized. It presents a new and powerful approach to treat or prevent many diseases including respiratory disorders by modulating gene expression [8,9,10,11,12,13]. RNAi can be mediated by various types of RNA molecules, including long dsRNA, short interfering RNA (siRNA), short hairpin RNA (shRNA) and microRNA (miRNA). The mechanism of RNAi is illustrated in Figure 1, and the general properties of different types of RNAi molecules are summarized in Table 1.

### 1.1. Long dsRNA and siRNA 

Long dsRNAs (around 500–1000 nucleotides in length) have been employed to study gene functions. After the exogenous dsRNA is introduced into the cytoplasm of the cells, it is cleaved by RNase III enzyme Dicer into short dsRNA called siRNA. The siRNA which is 21–23 nucleotides in length is loaded into a protein complex called the RNA-induced silencing complex (RISC). The siRNA is then unwound, and the sense strand (also known as the passenger strand) of the siRNA is degraded, whereas the remaining antisense strand (also known as the guide strand) guides the activated RICS to the target messenger RNA (mRNA) through full complementary binding. The mRNA is then cleaved by Argonaute 2 (Ago2) in the RISC, leading to the silencing of the target gene [14,15]. Since long dsRNA is known to trigger immunostimulatory response through the activation of Dicer-related antiviral pathways and induction of type 1 interferon (IFN) [16], it is less suitable for therapeutic use. In contrast, synthetic siRNA is a more promising gene silencing mediator because of the lower risk of immune response. It is also the most widely investigated RNAi molecule for therapeutic applications, with over 26 clinical trial studies being initiated since 2004 [17].

### 1.2. shRNA 

shRNA is a sequence of RNA transcribed in the nucleus of the cells from a DNA vector by either RNA polymerase II or III. The primary transcript is called primary shRNA (pri-shRNA), which contains a hairpin like stem-loop structure. The pri-shRNA is processed into a 50–70 nucleotides long loop-stem precursor shRNA (pre-shRNA) by a protein complex containing the RNase III nuclease Drosha and the dsRNA binding domain protein DGCR8. It is then transported to the cytoplasm through a specialized nuclear membrane protein, exportin-5 (Exp5). The loop sequence of the pre-shRNA is cleaved by the Dicer to form a double-stranded siRNA. This endogenously produced siRNA is loaded into RISC and induce RNAi through the similar process as the synthetic siRNA [18,19]. Since shRNA expression unit can be incorporated into viral vectors and continuously synthesized by the host cell, it can induce long-lasting gene silencing effect. The RISC-loading process of shRNA is about ten times more efficient than siRNA, implicating that lower dose of shRNA is required to maintain the therapeutic efficacy with less off-target effect [20]. However, the shRNA approach is a DNA-based strategy depending on the expression of shRNA encoding gene which often requires viral vectors. From the delivery perspective, the introduction of synthetic siRNA to the cytoplasm is a more straightforward way to induce RNAi. The use of viral vectors for delivery poses safety concerns in its therapeutic applications [15].

### 1.3. miRNA

miRNA is a naturally occurring non-coding RNA that plays a key role in regulating gene expression. Primary miRNA (pri-miRNA) is transcribed by RNA polymerase II from endogenous miRNA gene in the nucleus. The hairpin-containing pri-miRNA is structurally similar to the pri-shRNA, therefore the maturation pathway of miRNA is also similar to shRNA [18]. The pri-mRNA is converted by Drosha/DCGR8 complex into precursor miRNA (pre-miRNA), which contains 70–100 nucleotides with interspersed mismatches and adopts a loop structure. The pre-miRNA is subsequently transported to the cytoplasm by Exp5, and is processed by the Dicer into mature miRNA of 18–25 nucleotides in length [21]. As opposed to siRNA, the antisense strand of miRNA is only partially complementary to the target mRNA, leading to gene silencing via translational repression and/or mRNA deadenylation. Position 2 to 7 of the 5′ end of the miRNA is the “seed sequence”, which is essential for target recognition, and the miRNA binding sites of mRNA are located in the 3′ untranslated region (UTR) [22]. There are two major approaches of miRNA-based therapeutics: (i) miRNA inhibition [23,24], which suppresses the action of the endogenous miRNA by antisense oligonucleotide (antimiR); and (ii) miRNA replacement [25,26], which introduces synthetic miRNA (miRNA mimic) to restore the functions of the endogenous miRNA. Only the latter is discussed in this review. Compared to siRNA-based therapeutics, miRNA has the potential to target multiple genes due to the imperfect binding to the target mRNA [27]. 

## 2. Pulmonary Delivery of RNAi Therapeutics 

**Figure 2:**
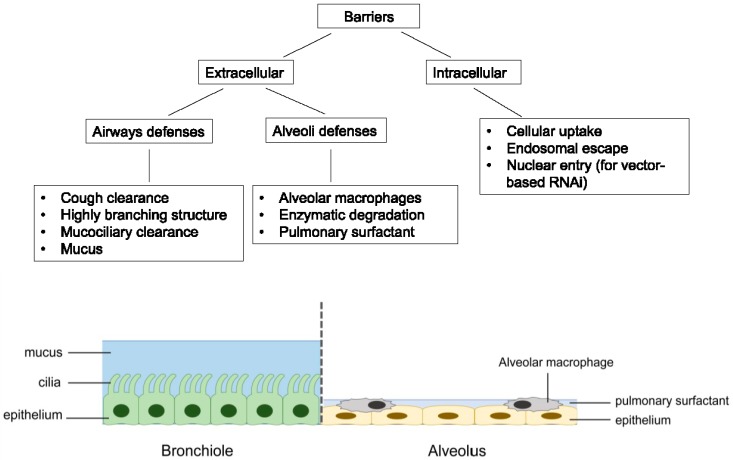
Summary of the major barriers associated with pulmonary delivery of RNAi molecules.

RNAi therapeutics offer several advantages over the traditional small molecules and protein-based drugs. They could virtually target any genes with high selectivity, including those “undruggable” targets. The design and synthesis of RNAi molecules are relatively simple because they do not need a cellular expression system, complex protein purification, or refolding schemes [28]. Compared to the antisense oligonucleotides, RNAi is more potent and specific [29]. Despite its huge therapeutic potential, delivery remains to be a major barrier to the clinical application of RNAi therapeutics [30,31]. For the treatment of respiratory disorders, pulmonary route of administration can deliver RNAi molecules directly to the site of action, thereby lowering the dose required while minimizing systemic adverse effects. The RNAi molecules can also avoid the rapid clearance by serum nuclease in the bloodstream. Moreover, inhalation is a non-invasive route of administration that is easily accepted by patients compared to parenteral administration, leading to better patient compliance [14,32]. However, pulmonary drug delivery is a challenge with several key biological barriers, which are highlighted in Figure 2.

### 2.1. Extracellular Barriers 

**Figure 3:**
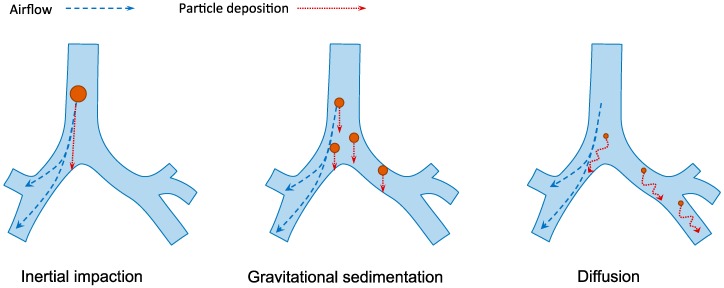
The three major mechanisms of particles deposition in the lungs. Inertia impaction occurs when large particles (>5 µm) cannot adjust to the change of airflow direction due to inertia and deposit on the airway walls, typically in the upper respiratory tract. Gravitational sedimentation is the settling of particles due to gravitational force. It mainly affects particles with size of 1–5 µm, which usually deposit in the small airways. Diffusion occurs when submicron-size particles having Brownian motion hit the surface of airway walls.

Human respiratory tract is divided into the conducting region (nasal cavity, pharynx, trachea, bronchi and bronchioles) and the respiratory region (respiratory bronchioles and alveoli). The former is the pathway for gases conduction whereas the latter is the site of gaseous exchange [14]. The extensive branched structure of the airways with varying length and diameter presents the primary hurdle in pulmonary drug delivery. The mechanisms of inhaled particle deposition in the lungs include inertial impaction, gravitational sedimentation, diffusion, interception and electrostatic precipitation [33]. As illustrated in Figure 3, the first three mechanisms are the major mechanisms which are greatly influenced by the aerodynamic size of the particles [34]. The optimal particle size for lung deposition is between 1 and 5 µm [35,36]. Larger particles are likely to be impacted and trapped on the upper airway wall at bifurcations while the small particles from 0.1 to 1 µm are easily exhaled during normal breathing. Smaller particles under 100 nm may be successfully deposited in the alveolar space because of the increasing diffusional mobility [37]. However, aerosols under 100 nm are difficult to produce, which make their application less favorable.

The presence of fluid layers in the airways, including the mucus and the pulmonary surfactant, also creates a barrier to the delivery of drug molecules to the lungs. Mucus is a gel of three-dimensional network structure. It acts as a sieve that filters out large molecules (>500 nm) [38]. Mucins, which are the major components of mucus, contain the hydrophilic glycosylated residues rich in serine and threonine and the hydrophobic non-glycosylated cysteine-rich domains. This characteristic allows electrostatic, hydrophilic and hydrophobic interactions between mucus and drug particles [39]. Since mucus is continuously produced, shed and replaced, and together with the mucociliary clearance action, the fast turnover rate of mucus leads to the rapid clearance of the entrapped drug molecules, preventing them from reaching the epithelium [40,41]. Moreover, cough clearance that occurs when the mucus reaches the throat is another removal mechanism of RNAi molecules from the airways. On the other hand, the role of pulmonary surfactant in nucleic acid delivery is controversial. Pulmonary surfactant is composed of approximately 90% lipids and 10% proteins [42]. It has been suggested that the lipids and proteins in the lung surfactant interact with the non-viral cationic lipid-based delivery system, leading to the premature release of nucleic acids [43,44]. Conversely, polymer-based delivery systems were found to be more compatible with pulmonary surfactant. Commercial pulmonary surfactant was employed in the preparation of some polymeric nanoparticles to facilitate the cellular uptake of siRNA to improve gene silencing effect [45,46].

Apart from the physical barriers, the RNAi molecules may undergo enzymatic degradation following pulmonary delivery. RNA is extremely susceptible to nuclease activity. Naked siRNA has a short half-life of less than 15 min in serum [47]. Due to the lack of serum in the lungs, the half-life of unmodified siRNA in the airways can reach up to three hours in mouse [48]. RNAi molecules may also be taken up and removed by alveolar macrophages upon lung deposition. The alveolar macrophages are responsible for engulfing and digesting foreign particles in the lower airways via phagocytosis. Unless the target of RNAi molecules is located inside the macrophages, such as *Mycobacterium tuberculosis* which is the causative agent of tuberculosis that typically reside in the alveolar macrophages [49], otherwise the RNAi molecules are subjected to degradation inside the macrophages before reaching their target sites.

### 2.2. Intracellular Barriers 

Once the RNAi molecules overcome the aforementioned extracellular barriers and reach the target cells, they need to overcome a set of intracellular barriers. The ultimate site of actions depends on the types of RNAi molecules. For synthetic siRNA and miRNA mimic, they need to reach the cytoplasm where the RISC locates, whereas DNA encoding shRNA or miRNA need to enter the nucleus in order for transcription to take place. 

Surface adhesion is the first step of cellular entry. There are various endocytic mechanisms involved in the cellular uptake of macromolecules, which subsequently influence their intracellular trafficking and delivery efficiency [50,51,52,53,54,55]. The clathrin-dependent endocytosis is the most common route of cellular entry for macromolecules [56]. Following cell entry, the RNAi molecules are entrapped in the early endosomes where progressive acidification occurs. The late endosomes then fuse with the lysosomes, which contain hydrolases that degrade the RNAi molecules [56,57]. The RNAi molecules must escape from endosome at early stage to exert their biological effect. Strategies of endosomal escape have been reviewed in other literatures [58,59,60,61]. For shRNA or miRNA vectors, nuclear entry is an additional barrier which is particularly challenging due to the presence of nuclear membranes that are impermeable to most substances except small non-polar molecules [62]. Non-viral vectors are inefficient in delivering DNA into the nucleus. Some viruses are evolved to transport their genome into the nucleus; hence, they become attractive DNA carriers to improve transduction efficiency. However, poor safety profile, high production cost and the risk of immunogenicity [63] limited the applications of viral vector mediated RNAi to laboratory tool. 

## 3. Delivery Strategies of RNAi Molecules

A delivery vector or carrier is generally necessary to overcome the aforementioned barriers by promoting cellular uptake and offering protection to the RNAi molecules. RNAi delivery system can be categorized into viral and non-viral vectors according to their nature, and these delivery systems have been extensively reviewed [63,64,65,66,67].

### 3.1. Viral Vectors

The viral vectors refer to the of use of viruses to deliver genetic materials to the cells. They are extremely efficient in transducing cells and providing either transient or long-term gene expression, depending on the type of viruses employed. Most of the in vivo studies used the adenoviruses, adeno-associated virus (AAV) and retroviruses to express the plasmid DNA encoding shRNA or miRNA to induce RNAi [68]. The most attractive property of viral vectors is that they have the ability to access the nucleus of the cells. Thus, they have high efficacy to express the RNA and subsequently regulate the gene expression [69,70,71,72]. However, the clinical application of viral vectors is limited by the toxicity, insertional mutagenesis (associated with retroviruses) and immunogenicity (associated with adenoviruses) [63]. In addition, the smaller size of AVV limits the amount of therapeutic gene that can be inserted. 

### 3.2. Non-Viral Vectors

Compared to viral vectors, non-viral vectors generally have better safety profile and lower production cost. They have been investigated for the pulmonary delivery of the RNAi molecules. Lipid-based, polymer-based and peptide-based are three major non-viral delivery systems [73]. The lipid-based vectors are the most commonly used vectors for RNAi delivery. They include cationic liposomes, solid lipid nanoparticles, solid nanostructured lipid carriers, lipidoids and pH-responsive lipids [74]. Many commercially available transfection agents (i.e., Lipofectamine, Oligofectamine, TransIT-TKO and DharmaFECT) are lipid-based systems [75,76,77,78]. Despite the promising transfection efficacy, the major challenges of using the lipids are the immune response and cytotoxicity [79]. In addition to the lipids, biocompatible and biodegradable natural polymers such as chitosan and dextran, as well as synthetic polymers such as poly lactic-co-glycolic acid (PLGA), polyethylenimine (PEI) and PAMAM dendrimer are used for the preparation of polymeric nanoparticles for RNA delivery [80,81,82,83,84,85]. The polymers have relatively low toxicity compared to the lipid-based vectors and are more versatile for chemical modification. For peptide-based delivery systems, peptides such poly(l-lysine) (PLL), cell penetrating peptides (CPPs) and pH-responsive peptides have been investigated for the delivery of the RNAi molecules [86,87,88,89,90]. Different types of peptides mediate cellular transfection via different mechanisms such as facilitating cellular uptake and promoting endosomal escape. Peptides can be used to carry the RNAi molecules alone or act as a functional component in other carriers. Currently, efficient peptides with low toxicity and high transfection efficacy are still under development. It is noted that for pulmonary delivery, the reduced nuclease activity in the lungs makes successful delivery of unmodified naked RNA become possible [91,92]. However, the mechanism of how naked siRNA could overcome the extracellular and intracellular barrier following pulmonary administration still remains to be understood. 

## 4. RNAi Therapeutics for the Treatment of Respiratory Diseases

The therapeutic potential of RNAi-based therapy targeting respiratory disorders has been widely explored in the past decade. In the following sections, the pre-clinical and clinical studies that involved the delivery of RNAi molecules to the airways for the treatment of lung diseases are discussed.

### 4.1. Lung Cancer 

Lung cancer is a leading cause of cancer death in the world. The mainstay of treatment is chemotherapy, which is indicated for around 70% of newly diagnosed patients with local and advanced metastatic disease [93]. Other treatment options include surgery and radiotherapy [94]. Angiogenesis inhibitors [95] and epidermal growth factor receptor (EGFR) inhibitors [96,97] are new medications licensed for the treatment of lung cancer. Despite various treatment options, the prognosis of lung cancer is poor. The major problems of chemotherapy are toxicity, lack of specificity and drug resistance [98]. In recent years, pulmonary delivery of RNAi molecules is being explored as a new treatment for lung cancer [99]. Genes that are associated with tumor cell growth and drug resistance are potential targets. Table 2 summarizes some recent in vivo studies of pulmonary RNAi delivery for lung cancer treatment. 

#### 4.1.1. Inhibition of Tumor Cell Growth 

Genes that are essential for tumor growth and progression, such as Akt1 (RAC-alpha serine/threonine-protein kinase B) and c-myc, are evaluated as RNAi targets. Akt1 is an important mediator of cell growth, proliferation and survival of non-small cell lung cancer (NSCLC). Activation of Akt1 pathway is observed in most NSCLC patients. It is found to reduce chemo- and radiation-induced apoptosis to extend the survival of tumor cells [100,101]. Sorbitol diacrylatepolyethylenimine (SDA-PEI) was employed to deliver shRNA targeting Akt1 (shAkt1) and cDNA encoding programmed cell death protein 4 (Pdcd4) in a dual expression vector via pulmonary route to murine lung cancer model. Simultaneous Akt1 inhibition and Pdcd4 overexpression could induce potent anticancer effect. The total tumor number and the tumor size larger than 1 mm in the lung were significantly reduced after eight doses [101]. Glycerol triacrylate–spermine (GT–SPE) polyspermine [102] and lentivirus [103] were also employed as carriers of shRNA targeting Akt1 signaling pathway for pulmonary delivery. Although repeated aerosol delivery of lentiviral-based shRNA can achieve potent knockdown of the target, inhalation is a procedure that generates large number of viral particles. There are safety concerns regarding the use of viral vectors for inhalation, including the potential mutagenicity, immunogenicity and the risk of aerosol-transmitted infections [104]. Therefore, aerosol delivery of RNAi molecules using viral vectors must go through vigorous biosafety evaluation for clinical use.

C-myc oncogene is a downstream conduit for most oncogenic signals. It is necessary for the growth control, differentiation and apoptosis. Abnormal expression of c-myc is associated with many tumors. De-activation of myc is efficacious for lung tumor therapy [111]. Conde et al. delivered siRNA targeting c-myc to the lung of mouse using RGD-surface-modified gold nanoparticles (AuNPs) by intratracheal instillation. RGD peptide (Arg-Gly-Asp) is a cell adhesion peptide responsible to mediate cell adhesion and proliferation by binding to the integrin avb3 receptor family, a specific marker for angiogenesis and up-regulated in the endothelium. This strategy successfully suppressed c-myc expression level and tumor cell proliferation, resulting in approximately 80% increase in survival of orthograft mouse tumor models [107].

#### 4.1.2. Targeting Multidrug Resistance (MDR)

Genes associated with MDR were also evaluated as target of RNAi, such as ribophorin II (RPN2) [106], B-cell lymphoma 2 (BCL-2) [112] and multidrug resistance protein 1 (MRP1) [110]. RPN2 is known to be associated with the apoptosis in docetaxel-resistant human breast cancer cells [113], and its involvement in NSCLC was investigated recently [114]. A novel RNAi molecule (PnkRNA) targeting RPN2 was developed and delivered to mice bearing lung cancer by intrapulmonary delivery. This new class of RNAi agent has a unique helical structure containing a central stem and two loops, and is claimed to be stable against nuclease degradation. Inhibition of lung tumor growth was observed without any signs of toxicity after treatment. The antitumor effect was triggered by PnkRNA targeting RPN2 without the use of other anticancer drugs. It could be attributed to the multiple activities of RPN2, which is anti-apoptotic and involved in the regulation of tumor survival [106]. However, the mechanism of how the naked the PnkRNA molecules overcame the extracellular and intracellular barriers to initiate RNAi was not elucidated.

Besides the inhibition of MDR associated proteins alone, the co-delivery of anticancer drugs with RNAi therapeutics is as an alternative approach to tackle MDR in various types of cancers [112,115,116,117]. Combination therapy offers the potential advantages of synergistic activity, overcoming the drug resistance and reducing the side effects of chemotherapy. Bcl-2 is a main player of nonpump cellular resistance [118] and MRP1 is responsible for drug efflux in cancer cells [119]. They were investigated in the combination approach. Taratula et al. prepared a lipid-based nanoparticles containing siRNA targeting Bcl-2 and MRP1, as well as anticancer drugs (doxorubicin or paclitaxel). The nanoparticles were delivered to the lung of tumor mouse model using a nose only exposure chamber for inhalation. Almost complete disappearance of lung tumor was observed in animals treated with paclitaxel plus the siRNAs. In contrast, treatment with paclitaxel alone only slowed down the growth of the tumor [110]. Xu et al. developed a polyethylenimine (PEI)-based delivery system that simultaneously delivered siRNA targeting Bcl-2 and doxorubicin to the lungs of mice with metastatic lung cancer. Synergistic antitumor efficacy was achieved compared with the delivery of doxorubicin or siRNA alone [105]. These studies indicated that suppression of resistance gene by RNAi molecules via inhalation could restore the sensitivity of chemotherapeutic drugs, leading to the death of tumor cells.

### 4.2. Respiratory Infection

Respiratory infection can be caused by a wide range of microorganisms in the airways including bacteria and viruses [120]. It is one of the most common reasons for hospitalization among adults [121]. Due to the rise of antimicrobial resistance, many infectious diseases including respiratory infections have become difficult to treat. RNAi therapeutics appears to be an attractive approach to fight against infections [122]. The pre-clinical studies of RNAi therapeutics to combat against respiratory infections are summarized in Table 3.

Since RNAi was first reported to inhibit respiratory syncytial virus (RSV) in 2001 [131], an increasing number of studies have been initiated to explore the potential of RNAi technology to treat other respiratory viral infections including influenza [86,127,132,133] and severe acute respiratory syndrome (SARS) [134]. RNAi molecules offer several advantages as antiviral therapeutics. They can specifically target the conserved region of mRNA sequence in the viral genome, making it effective against mutated viral strains. In addition, RNAi molecules can be designed rapidly to target viral genes, shortening the lead time of developing new antiviral agents, which is particularly useful in response to an infection outbreak. To achieve effective antiviral effect, the viral targets must be essential for the pathogenesis of viral infection and/or replication cycle. It is also desirable that the target genes share similar conserved sequence among different strains to achieve broad viral inhibition. Furthermore, the viral gene should be substantially different with human gene to minimize any undesirable side effects.

#### 4.2.1. RSV Infection

RSV infection is one of the most common infections in children [2,135]. In adult, RSV infection usually leads to self-limited upper respiratory illness [136]. However, severe RSV infection has been observed in elderly patients with lung diseases or in immunocompromised patients [137]. Current management of RSV includes bronchodilators, corticosteroids, antibiotics and supportive care [2], but their efficacy is not satisfactory. To date, there is no licensed vaccine available to prevent this disease [138]. There is an unmet need to develop effective RSV therapeutics and vaccines in both the pediatric and adult population.

In 2005, Bitko et al. reported the inhibition of RSV and parainfluenza virus (PIV) replication by siRNA targeting viral phosphoprotein (P protein), a crucial subunit of the viral RNA-dependent RNA polymerase. Since RSV replicate primarily in the superficial layer of the respiratory epithelium, local delivery of RNAi molecules to the lungs is a rational approach to inhibit RSV replication [139]. In the study, TransIT-TKO was employed as transfection agent and the siRNA was administered to the lungs of BALB/c mice by intranasal instillation 4 h before viral challenge. The RSV and PIV titer was reduced by over 90% five days after infection. The use of naked siRNA also showed substantial inhibition of infection, with approximately 70%–80% efficiency achieved as compared to complexed siRNA. Interestingly, the administration of siRNA before and concomitant with RSV infection was more effective in reducing viral load than treatment after infection [123]. The prophylactic regimen may not be a clinically attractive approach. To achieve optimal therapeutic benefit, fast detection and early intervention are crucial for the prognosis of RSV infection. It is desirable to initiate the antiviral RNAi therapy as quickly as possible once RSV infection is confirmed in clinical practice. 

P protein may not be an ideal target as it is limited by its specificity to a particular viral strain [124]. Another siRNA, ALN-RSV01, was designed to target the highly conserved region of the mRNA encoding viral nucleocapsid protein (N protein). N protein is a core protein in the RNA polymerase and plays a key role in the replication cycle of RSV [140]. After intranasal administration of ALN-RSV01 to the lungs of mouse at 4 h prior to viral infection, 2.5- to 3.0-log-unit reductions in viral load was observed in comparison with the mismatch siRNA control. For the treatment regimen, comparable antiviral efficacy was achieved in multiple daily doses of ALN-RSV01 at day 1, 2 and 3 post-infection [124]. 

The phase I clinical study of ALN-RSV01 was reported in 2008 [139]. Intranasal administration of ALN-RSV01 was well tolerated and the side effect profile was similar to placebo. Later in 2010, the antiviral effect of ALN-RSV01 was evaluated in a phase II trial. ALN-RSV01 was administered by nasal spray daily to healthy adults for two days before and for three days after RSV inoculation. The group treated with ALN-RSV01 had a significantly lower number of RSV infection compared to the placebo group [140]. Another phase II b clinical trial of ALN-RSV01 in RSV-infected lung transplant patients was also completed [141]. The primary endpoint of the study was to evaluate the effect of ALN-RSV01 on the incidence of new or progressive bronchiolitis obliterans syndrome (BOS) in RSV-infected lung transplant patients. Even though the study marginally missed the primary endpoint statistically (*p* = 0.058), inhaled ALN-RSV01 treatment induced a clinically meaningful reduction in the incidence of BOS, reinforcing the potential of inhaled RNAi as new antiviral therapeutics.

#### 4.2.2. Influenza

Seasonal influenza viruses, especially influenza type A virus, cause annual epidemics that led to millions of death worldwide [142]. It is one of the major public health problems. Two categories of antiviral drugs are currently approved to treat influenza, including M_2_ ion-channel protein inhibitors (adamantanes) and neuraminidase inhibitors (zanamivir and oseltamivir). However, some influenza A virus strains have shown to be resistant to these drugs [143,144], making the development of new antiviral drug urgently needed. A number of studies were carried out to investigate the use of RNAi to inhibit viral gene expression and protect cells from influenza infection. The most commonly investigated targets are the nucleocapsid protein (NP), polymerase acidic protein (PA) and polymerase basic protein (PB). These proteins are indispensable to influenza viral replication. More importantly, they are highly conserved across different subtypes of influenza virus strains [132]. 

The first animal study applying RNAi against influenza was reported by Ge et al. [145]. siRNA targeting NP or PA were complexed with PEI. The complexes were injected intravenously to the mice 3 h before or 5 h after influenza virus (PR8 H1N1) infection. The antiviral effect of siRNA targeting NP was dose-dependent and combination of siRNAs targeting NP and PA enhanced the effect. In the same study, shRNA targeting NP or PB was also investigated. The DNA was mixed with Infasurf, a commercial available pulmonary surfactant. After intranasal instillation, a significant reduction of virus titer was detected 24 h after infection although the effect was less prominent compared with intravenous injection of PEI complexes. Infasurf may play a role as nucleic acid carrier. Little is known about the interaction between DNA and pulmonary surfactant in the airspace. It could be possible that the DNA interacted with the charged lipid components in the pulmonary surfactant leading to the efficient spread and absorption in the airways [146]. 

Another study reported the administration of siRNA targeting NP and/or PA in lipid carrier to influenza-infected mice by hydrodynamic injection and intranasal administration. The combined siRNA delivery method effectively protected animals against lethal challenge of highly pathogenic avian influenza A viruses, including the H5N1, H7N7 and H9N2 subtypes [126]. Since then, the potential of siRNA for the treatment of influenza virus infection was continuously evaluated in vitro [133,147,148,149] and in vivo [127,128,129]. However, the exploration of RNAi for the treatment of influenza appears to reach the bottleneck. It could be partly attributed to the criticism of the antiviral effect induced by siRNA, which was due to innate immune response rather than viral inhibition through RNAi. Development of chemically modified RNAi molecules with minimal immunostimulatory effect could be helpful to delineate the mechanism of the antiviral effects [150]. 

#### 4.2.3. Tuberculosis 

Tuberculosis (TB) is a bacterial lung infection caused by *Mycobacterium tuberculosis* (Mtb). Because of the emergence of multidrug-resistant (MDR) and extensively drug-resistant (XDR) TB, the slow development of new anti-TB agents and the inefficiency of vaccination, TB is still a major global health problem. WHO reported that 9.6 million people suffered from TB in 2014 and 1.5 million die annually because of the disease [151]. A new and effective TB therapeutics is highly sought after.

The approach of RNAi-based therapeutics against TB aims to modulate the gene expression of the host instead of the bacillus because bacteria do not contain the requisite machinery for RNAi. Currently, RNAi has been investigated for TB treatment in two directions: (i) targeting the host factors that are essential for the survival of mycobacteria; and (ii) modulating the host immune response to facilitate the elimination of mycobacterium [49]. Autophagy is a host defense mechanism against Mtb. Boosting autophagy could be an effective strategy to promote the eradication of the mycobacteria. Hence, a number of host factors that are involved in the regulation of autophagy were explored as the target of RNAi, including Bfl-1/A1 [152], Rap22a [153], Ras homologue enriched in brain (Rheb) [154] and UV radiation resistance-associated gene (UVRAG) [155]. Down-regulation of these factors promoted autophagosome formation and reduced the bacterial growth. However, the existing studies of using RNAi to suppress host factors are preliminary and in vivo data is lacking. Moreover, it was recently reported that autophagy pathway may not be essential for restricting Mtb growth [156]. Further investigations are required to understand the relationship between Mtb infection and autophagy pathway, and to better position the approach of RNAi for the control of tuberculosis infection. 

Another strategy is to modulate host immune response to improve the antimicrobial ability of the host [49]. One of the key characteristics of TB infection is the formation of granuloma, which is a compact, organized aggregate of immune cells. Granuloma is previously considered as a host-protective structure to sequester the infecting mycobacteria, but recent work revealed that the pathogens could take advantage of granuloma formation to facilitate their proliferation and dissemination in the host [157], as it provides a hospitable environment for mycobacteria to survive and replicate over a long period of time. There is a complicated chemokines and cytokines network involved in the granulomatous response. RNAi can be used to target immunosuppressive cytokines in order to inhibit the growth of Mtb. Among them, tumor necrosis factor (TNF)-α and IFN-γ are critical in activating macrophages and triggering the formation of granuloma [158]. XCL1 (lymphotactin) is a chemokine produced by activated CD8^+^ T cells during chronic TB infection. It was suggested that XCL1 regulates IFN-γ production by CD4^+^ T cells and contribute to the stability of the granuloma [159]. When a single dose of siRNA targeting XCL1 was delivered to the lungs of mice that were chronically infected with TB, the expression of XCL1 was effectively inhibited. The suppression of XCL1 expression in the lungs was associated with the decreasing number of T lymphocytes, reduction in the IFN-γ response, disorganized granulomatous lesions and higher fibrosis [130]. Although the inhibition effect elicited by siRNA was transient and did not provide significant therapeutic benefit, this proof-of-concept study demonstrated that the local-environment and immunopathology of the lungs can be modulated to favor host response via pulmonary delivery of siRNA. 

Other targets of RNAi-based immunotherapy include the transforming growth factor β (TGF-β) and interleukin 10 (IL-10) [13]. Both of them are found to be elevated in TB patients, and their high level of expression is connected with TB reactivation [160]. During chronic infection, these cytokines restrain inflammation through affecting helper T cell 1 (T_h_1) response and macrophage activation, leading to substantial reduction of their antimicrobial activity [161]. In that study, siRNA targeting TGF-β1 was administered through the intrapulmonary route to IL-10 knockout mice at 60 days post-infection. TGF-β1 gene silencing resulted in the increased expression of the antimicrobial mediators nitric oxide (NO) and inducible nitric oxide synthase (iNOS), and significantly reduced the bacterial load for at least four weeks after treatment. A synergistic effect at improving the antimicrobial activity in the lungs was observed when both IL-10 and TGF-β1 were targeted simultaneously. Although the inhibition of bacterial load in this study was modest, it showed that siRNA immunotherapy is a feasible approach to enhance the host antimicrobial capacity. Moreover, MDR and XDR TB pose huge challenges for the current chemotherapeutic agents. Combined immunotherapy targeting immunosuppressive cytokines and chemotherapy to target drug resistance bacteria population is a direction that is worth future investigation.

### 4.3. Respiratory Inflammatory Disease 

Respiratory inflammatory diseases impose substantial healthcare burden worldwide as many of the patients are inadequately controlled by current therapies [4,5,162]. These diseases are characterized by inflammation in the respiratory tract, which is manifested with increased expression of inflammatory cytokines and chemokines [163,164]. The common lung inflammatory diseases include asthma, chronic obstructive pulmonary disease (COPD) and acute lung injury (ALI). Table 4 lists the major in vivo studies that examine the effects of RNAi molecules in respiratory inflammatory disease.

#### 4.3.1. Asthma

Asthma is characterized by the variable and reversible airflow obstruction, airway hyperresponsiveness (AHR), mucus hypersecretion and chronic inflammation [176]. The most common form of asthma is allergic asthma, which is triggered by environmental stimuli such as house dust and seasonal pollen [177]. According to WHO, it is estimated that around 334 million peoples have asthma worldwide [178]. The mainstay treatments of asthma are corticosteroids, β_2_-adrenergic receptor agonists and leukotriene receptor antagonists, either alone or in combination. Although these medications can manage the conditions in most of the patients, there are still challenges of poor patient compliance, intolerability and long term adverse effects [179]. For persistent allergic asthmatic patients, omalizumab which is an anti-immunoglobulin E (IgE) monoclonal antibody can be used, but the high cost and parenteral administration make it less favorable [180]. Alternative anti-inflammatory drugs are needed. Many inflammatory mediators are identified to be involved in the pathogenesis of asthma and they are investigated as potential targets of RNAi therapy. These include cytokines/chemokines [181], transcription factors [168], tyrosine kinases/tyrosine kinase receptors [172] and co-stimulatory molecules [182].

In allergic asthma, there is predominant activation of CD4^+^ T helper 2 (T_h_2) cell-driven inflammatory response [183]. T cell activation and T_h_2 cytokine expression are increased in the airways of atopic asthma patients after exposure to allergen [184]. T_h_2-type cytokines, interleukin-4 (IL-4), IL-5 and IL-13, are the main players in the pathology of allergic airway inflammation, contributing to allergic sensitization, IgE production, eosinophil infiltration and AHR respectively [183,184]. Clinical studies that examine the therapeutic potential of using monoclonal antibodies (mAbs) to neutralize the bioactivity of IL-4 [185], IL-13 [186,187] and IL-5 [188] or target their receptors are in progress. Similarly, RNAi technology can be employed to target these cytokines [169,181]. 

Targeting suppressor of cytokine signaling (SOCS) 3 is an alternative approach [12]. SOCS proteins are negative regulators of cytokine signaling pathway, which are involved in T_h_2 cells mediated allergic response via controlling the balance between T_h_2 and T_h_1 cells [189]. The expression of SOCS3 is a pathological marker of allergic disease. After siRNA targeting, SOCS3 was intranasally administered to the lungs of chronic asthmatic mouse model [12], the silencing of SOCS3 down-regulated the expression of T_h_2 cell associated cytokines, IL-4, IL-5 and IL-13, leading to substantial reduction of airway inflammation, AHR as well as IgE production. Reduction of mucus secretion and collagen deposition was also detected in the airways. Besides T_h_2 cell, other T cells subsets, such as T_h_9 and T_h_17 cells, also contribute the inflammatory response in asthma by releasing IL-9 and IL-17A. These molecules may also be the potential targets for RNAi therapy [183]. Furthermore, the combination of inflammatory and antiviral siRNAs is a prospective therapy for patients with virus-induced exacerbation of severe uncontrolled asthma, in view of the report that respiratory viral infection is related to 80% of asthma exacerbation in children and adults [190]. The therapeutic potential of targeting IL-4 and P protein of RSV simultaneously by siRNAs was examined in a mouse model of RSV-induced asthma exacerbation [181]. The intranasal delivery of the two siRNAs was able to: (i) significantly suppress IL-4 mRNA expression and RSV replication in the lungs; (ii) reduce eosinophil and neutrophil infiltration in bronchoalveolar lavage fluid (BALF); (iii) inhibit AHR and inflammation; and (iv) increase interferon (IFN)-γ expression. 

Many transcription factors including nuclear factor-κB (NF-κB) [168], signal transducer and activator of transcription factor 6 (STAT6) [166] and GATA-binding protein 3 (GATA3) [170] are involved in the production T_h_2 cytokine in the airways of patients with asthma. They were investigated as targets for RNAi in the management of asthmatic inflammation. NF-κB plays a key role in the expression of many pro-inflammatory proteins [191]. Inhibition of NF-κB pathway in the lungs was reported to be beneficial in asthma model [176,192]. One approach to inhibit the activation of NF-κB signaling pathway is to target receptor-interacting protein 2 (Rip2), which is a transcriptional product and an activator of NF-κB. Suppression of Rip2 expression was achieved in mice by intratracheal delivery of siRNA, resulting in the inhibition of ovalbumin (OVA)-induced cytokine release, inflammatory cell infiltration, mucus hypersecretion and serum IgE level. This result suggested that Rip2 could be a novel therapeutic target for the treatment of asthma [168]. Consistent with this finding, suppression of STAT6 [10] and GATA3 [170,193] in murine model of allergen-induced asthma by RNAi significantly inhibited the expression of downstream T_h_2 cytokines. These studies support the hypothesis of using RNAi molecules targeting transcription factors to manage airway inflammation and AHR of asthma. 

Tyrosine kinases, which are broadly classified into receptor and non-receptor types, play an important role in activating the transcription factors for inflammatory gene expression upon activation by the inflammatory signals [194,195]. The key receptor tyrosine kinases involved in the inflammatory response in asthma include epidermal growth factor receptor (EGFR), c-kit (a stem cell factor receptor) and vascular endothelial growth factor receptor (VEGFR), while the non-receptor forms are spleen tyrosine kinase (Syk), Src and janus kinase (JAK) [196]. Syk is a pivotal intracellular signaling molecule involved in the activation of several pro-inflammatory transcription factors [197]. Inhibition of Syk expression by antisense oligonucleotides has proven effective in suppression of tracheal contraction and pulmonary inflammation in animal models of OVA-induced asthma [198]. When naked siRNA targeting Syk was administered to the lungs of allergen-induced asthma mice model via nasal instillation, the recruitment of inflammatory cells in the BALF was reduced [194]. Similarly, intranasal administration of siRNA targeting c-kit also induced a promising result in reducing mucus secretion and airway inflammation in experimental asthma mouse model [173]. Moreover, clinical studies have been performed on a siRNA targeting Syk (Excellair™ developed by ZaBeCor Pharmaceuticals, USA) for the treatment of asthma. In the Phase I study, 75% of asthma patients receiving Excellair™ by inhalation for 21 consecutive days showed improvement in breathing or reduced the usage of rescue inhaler compared to placebo. The drug was well tolerated by patients without eliciting serious side effects. A Phase II clinical trial Excellair™ has been initiated in 2009; the results are not yet available during the preparation of this review [199,200].

Targeting dendritic cells (DCs) in the airways by RNAi is another strategy for the treatment of asthma. DCs are antigen presenting cells that could be found in the airways [5,201]. They are actively involved in the differentiation of T_h_2 cells and hence contribute to the progress of airway inflammation [202]. DCs express co-stimulatory molecules, cluster of differentiation (CD) 80 and 86, on the surface. The binding of CD80/CD86 with CD28 and cytotoxic T lymphocyte-associated antigen-4 (CTLA-4) molecules on T cells is necessary for priming naive T cells into T_h_2 cells [203]. Blocking CD80 and/or CD86 activity with pharmacological inhibitors can inhibit T cell activation and alleviate AHR and pulmonary inflammation in mice exposed to aerosolized allergen challenge [204]. Likewise, intratracheal administration of siRNA targeting CD86 significantly reduced its expression in the airway mucosa of mouse model of allergic asthma. CD86 siRNA treatment also contributed to the amelioration of OVA-induced airway eosinophilia, hyperresponsiveness, the elevations of IgE in the sera and the production of inflammatory cytokines in BALF [171]. 

#### 4.3.2. COPD

COPD is a persistent lung disease characterized by irreversible chronic airflow limitation. The disease is usually progressive and associated with an increased chronic inflammatory response in the lungs [205,206]. It is the result of long term exposure to air pollutants and usually associated with cigarette smoking [205]. It affects around 10% of the population in the world. It is the fourth leading cause of death in most of the industrialized countries and expected to become the third by 2020 [207]. Three classes of medications used to manage COPD are bronchodilators (including β_2_-adrenergic receptor and anticholinergic drugs), corticosteroids and phosphodiesterase-4 inhibitor. They are effective in relieving the symptoms of COPD, but none of them can reverse the progressive deterioration in lung function or cure the disease [4,205]. It is desirable to develop a novel therapy that can simultaneously control the symptoms and improve the long-term prognosis of the disease. 

Despite similar clinical features, the mechanisms of underlying airway inflammation in asthma and COPD are different with distinct patterns of inflammatory cells and mediators being involved [163]. The mechanisms and the mediators that drive the progression of chronic inflammation and emphysema in COPD are far from fully understood [208]. The major signaling factors involved in the pathogenesis of COPD include TNF-α, CCL2, CXCL8, TGF-β and VEGF [206]. Many of the cytokines and chemokines secreted in the airways of COPD patients are modulated by the NF-κB pathway [163]. Therefore, RNAi targeting NF-κB pathway can be a strategy for the management of COPD [176,209]. 

Current research work on RNAi therapy for COPD remains mainly in the in vitro stage, likely due to the insufficient understanding of the highly complex mechanisms underlying the pathology of the disease [120]. Moreover, the degree of airway inflammatory response is determined both by genetic and epigenetic factors. Since multiple inflammatory mediators are involved in the disease process, blocking a single type of molecule is unlikely to exert significant clinical impact [206]. miRNA has the advantage of targeting multiple genes simultaneously. It is involved in the regulation of the inflammatory process of COPD by modulating the transcription and translation of genes. Differentiated expression of several miRNAs, like let-7c, miR-125b, miR-15b [210] and miR-199a-5p [211] were identified in COPD patients compared to subjects without COPD. Modulation the expression of multiple genes using miRNA could be a direction of RNAi therapy for the treatment of COPD.

#### 4.3.3. Acute Lung Injury (ALI)

ALI is a severe form of diffuse lung disease that is commonly caused by acute systemic inflammatory disorders, such as sepsis, pneumonia, trauma and pancreatitis [11,212]. It is a progressive disease, which can lead to acute respiratory distress syndrome (ARDS) or more critically, multiple organ failure and even death [175]. Its pathological changes include the release of pro-inflammatory cytokines and chemokines, and the destruction of the epithelium-capillary interface; the latter can facilitate the extravasation of protein-rich fluid and the infiltration of neutrophils, thereby exacerbating the condition [213,214]. Since the molecular mechanism underlying the pathology of ALI is not well understood, there is no pathophysiologic-driven therapeutic approach available [162].

RNAi targeting the essential inflammatory mediators could offer protection against the progression of the disease. In an in vivo study, systemic delivery of siRNA targeting TNF-α significantly reduced the expression of TNF-α in the lungs and lung injury in a shock-induced ALI mouse model [175]. The findings suggested that pulmonary vascular cells, but not the epithelial cells, contribute to the release of TNF-α leading to lung injury following a systemic inflammatory insult. Although these findings are contradictory to the others who suggest that pulmonary epithelial cells are the major players for causing lung injury induced by a systemic disease [215,216], nevertheless, they provide evidence that support the use of siRNA targeting TNF-α in the management of ALI.

Increase microvascular permeability is a major cause of amplification of the inflammatory responses in ALI, leading to the progression to severe pathological condition [214]. The integrity of pulmonary and systemic endothelial barrier is maintained by the bioactive lipid, sphingosine-1-phosphate (S1P), which can be irreversibly degraded by an enzyme called sphingosine-1-phosphate lyase (S1PLyase) [214]. Therefore, suppressing endothelial S1PLyase expression by siRNA is a potential therapeutic approach to maintain the integrity of the endothelial barrier [217]. Nanoparticles containing siRNA targeting S1PLyase and recombinant high mobility group box-1 A peptide (HMGB1A) were delivered to the lungs of LPS-induced ALI mouse model by intratracheal instillation. HMGB1A peptide is an antagonist of the pro-inflammatory cytokine HMGB1 and provides additional anti-inflammatory effect in ALI. An arginine-rich R3V6 peptide was used as a carrier. S1PLyase level was significantly inhibited in the BALF, leading to a significantly reduction of inflammatory cytokine (TNF-α and IL-6) and inflammatory response in the lungs of the treated animal [11].

#### 4.3.4. Considerations of Managing Respiratory Inflammatory Diseases by RNAi 

Although several in vivo studies demonstrated promising results of using RNAi therapeutics for the management of respiratory inflammatory diseases, there are several concerns that need to be taken into consideration with this approach. The distribution of naked siRNA following intranasal and intratracheal administration was investigated. It was shown that the siRNA molecules were predominantly accumulated in the peribronchial epithelial cells within 24 h post-administration [12]. Since the inflammatory mechanism of diseases like asthma appears to be driven by the activated T_h_2 lymphocytes [218], it is highly desirable for RNAi molecules to target the activated T cells in the airways to achieve therapeutic benefit. However, specific targeting to T cells is extremely difficult [174]. Effective T cells transfection in vitro was achieved by electroporation [219], an approach that is not clinically feasible. Recently, a ligand-polymer conjugate siRNA delivery system, transferrin-polyethylenimine (Tf-PEI), has been developed to target T cells in the lungs [174]. The rational of such a design was based on the increased expression of transferrin receptors on T cells after activation. Biodistribution study showed that Tf-PEI polyplexes can selectively deliver the fluorescently labeled siRNA to the activated T cells in a murine asthma model. However, the therapeutic effect of the system was not evaluated. Therefore, targeting delivery of RNAi therapeutics to specific cell types associated with the disease is a field that certainly required further exploration.

Most of the key signaling molecules involved in the pathogenesis of respiratory inflammatory disorders have multiple functions and participate in normal physiological activities to maintain homeostasis. Short-term regimen (up to four days) was employed in the majority of the animal studies. The duration of treatment may not be sufficient to observe any long-term effect. Since many inflammatory diseases are chronic disorders, long-term therapy is often required to control the disease. Therefore, it is crucial to evaluate the long-term effect of any RNAi therapeutics.

Inhaled corticosteroid is commonly used to control the symptoms of lung inflammatory disorders. Steroid treatment reduces airway inflammation, partially through down-regulation of the key effectors molecules, such as c-kit [220] and GATA3 [221]. For this reason, it is necessary to determine the effect of concurrent medications on the expression of targeting molecules before initiating the RNAi therapy. Given that lung inflammatory diseases are heterogeneous diseases that are categorized into different subtypes according to the inflammatory profiles and responses to treatment, it is desirable to identify the patients’ profile in order to maximize the benefit of RNAi therapy.

### 4.4. Pulmonary Fibrosis

Pulmonary fibrosis is a respiratory disorder characterized by the progressive and irreversible destruction of lung architecture, which ultimately leads to organ malfunction, disruption of gas exchange and even death [222]. It is initiated by lung tissue damage resulting from autoimmune disorders or exposure to external irritants. The damage in turn triggers inflammatory reactions for tissue repair in the attempt to restore the normal tissue architecture and functions [222]. Under pathological conditions, extracellular matrix (ECM) components, which are released by the activated myofibroblasts during the wound healing process, are excessively produced and accumulate at the site of tissue injury, resulting in fibrosis [223]. There are many forms of pulmonary fibrosis. All of them are lethal diseases with high mortality rate but with very limited therapeutic options [223]. Thus pulmonary fibrosis is a lung disease with huge unmet medical need.

RNAi has been exploited to suppress the production of key molecules involved in the fibrotic process, such as TGF-β [92,224], amphiregulin (AR) and connective transforming growth factor (CTGF) [8,225] (Table 5). Clinical trials examining the effect of neutralized antibodies to block the activity of TGF-β are currently ongoing [14]. Since lung epithelial cells are the key cell types in the pathogenesis of pulmonary fibrosis, local administration of RNAi targeting TGF-β to the lungs can ensure high local concentration of RNAi molecules to produce therapeutic effects. Intratracheal delivery of siRNA targeting TGF-β1 could effectively inhibit pulmonary fibrosis, improved lung function, and prolonged survival in human TGF-β1 transgenic mice [224]. In another in vivo study, intranasal delivery of miRNA-326 mimic, which was designed to suppresses the expression of TGF-β and other profibrotic genes, significantly reduced TGF-β1 expression level and further alleviated the fibrotic feature in the bleomycin-induced pulmonary fibrosis [92]. The results suggest the potential of targeting TGF-β or its signaling pathway using RNAi technology for the management of lung fibrosis.

One complication of using TGF-β as the molecular target of RNAi is related to its physiological role in maintaining immune and cellular homeostasis. It has a role in tumor suppression as suggested by the finding that specific inhibition of TGF-β in mouse stromal fibroblasts results in spontaneous carcinoma formation in the adjacent epithelium [226]. As a result, targeting the downstream effectors of TGF-β may offer the therapeutic benefit of inhibiting the TGF-β-mediated fibrotic response while preserving the major physiological function of the profibrotic cytokine [227]. CTGF is a key molecule that regulates fibroblast mitosis and promotes collagen deposition in pulmonary fibrosis induced by TGF-β. A non-viral delivery system consists of poly(dimethylamino)ethylmethacrylate (PDMAEMA) and its copolymer with poly(methylether-methacrylate-ethyleneglycol) [PMAPEG] was used to incorporate and form complexes with siRNA targeting CTGF. After intratracheal administration, significant reduction of CTGF expression, collagen deposition and inflammatory cytokines (IL-6, TNF-α and TGF-β) production were observed in bleomycin-induced lung fibrosis animal model. More importantly, the survival rate of the animals treated with siRNA targeting CTGF was significantly increased in comparison with the group treated with scrambled siRNA [225]. 

Another downstream mediator of TGF-β is amphiregulin (AR), which is an epidermal growth factor receptor (EGFR) ligand primarily induced by TGF-β1 [228]. siRNAs targeting AR and CTGF were delivered to the lung of pulmonary fibrosis mouse model via either intratracheal or intravenous administration. To improve the efficiency and cell-specific delivery, a modified self-assembled micelle interfering RNA nanoparticles (SAMiRNA) was developed. The nanoparticles contain hydrophilic polymer and hydrophobic lipid on each ends of siRNA and can spontaneously form micelle in solution. After administration of AR or CTGF SAMiRNAs, the expression of AR and CTGF, and collagen accumulation in the lung of pulmonary fibrosis model were significantly reduced compared to control SAMiRNA [8].

## 5. Clinical Translation and Future Perspectives

RNAi technology has been under rapid development for the last decade. The therapeutic potential of RNAi molecules in airway diseases has been demonstrated in several clinical studies (Table 6), and they could be the solution to some unmet medical needs. No RNAi therapeutics has gained approval by regulatory authority at the time this manuscript was prepared. While delivery is generally considered as the major obstacle to RNAi therapeutics development, it is interesting to notice that siRNAs or miRNA mimics do not need a carrier to generate therapeutic effects following pulmonary delivery. The cellular uptake mechanism of naked RNA in the airways is not clear, but this is certainly an attractive feature that allows simple formulation without the need of delivery vectors. Chemically modified siRNA or miRNA is necessary to improve the stability of the RNA molecules. On the other hand, the delivery of shRNA or miRNA encoding plasmids requires viral vectors to ensure efficient transduction and the subsequent expression of RNAi molecules. However, the use of viral vectors could raise safety concerns for clinical applications, especially when they are used for pulmonary delivery. The employment of liposomal or polymer based nanoparticle delivery technology could be a promising approach. Whether the nanoparticles or naked RNA are used, it is important to evaluate the pharmacokinetic profile of each delivery system to ensure the RNAi therapeutics are localized at the site of action but not quickly distributed to other organs in the body. In terms of formulation of RNAi therapeutics, dry powder aerosol is preferred over the liquid aerosol due to higher stability and the dry powder inhalers are easier to operate with better lung deposition [229]. This would be the direction for RNAi formulation development.

Among the different types of RNAi molecules for therapeutic development, siRNA has made significant progress, with a couple of clinical studies investigate the pulmonary delivery of siRNA to treat respiratory diseases. There are also a growing number of clinical pipelines involving the use of siRNA and other RNAi molecules. Several key questions are raised that need to be answered. For infectious diseases, as demonstrated in several in vivo studies, it appears that RNAi is more effective in prophylactic regimen than in therapeutic regimen. How can we improve the therapeutic efficacy of RNAi in combating infections to make it clinically relevant? For inflammatory diseases, long-term therapy is expected. How can we minimize the undesirable effects when cytokines/chemokines are being targeted? When the respiratory functions are compromised in patients with lung disease, is pulmonary delivery a suitable route for administration? As complicated signaling pathway is involved in the pathogenesis of different lung disorders, a thorough investigation of the underlying pathological mechanisms of the disease is a prerequisite for identifying the target of RNAi. Despite the challenges and obstacles, we believe that the pulmonary delivery of RNAi molecules targeting airway diseases is promising. 

## Figures and Tables

**Table 1:** Comparison of general properties between long double-stranded RNA (dsRNA), short interfering RNA (siRNA), short hairpin RNA (shRNA) and microRNA (miRNA).

Properties	Long dsRNA	siRNA	shRNA	miRNA
Structure	ds, 500–1000 nt duplex	ds, 21–23 nt in length with 2 nt 3′ overhang	50–70 nt with a stem-loop structure (pre-shRNA), cleaved by Dicer to give RNA duplex with structure similar to siRNA	pre-miRNA: 70–100 nt with a stem-loop structure containing bulges/mismatches miRNA: 18–25 nt in length with 2 nt 3′ overhang
Synthesis	Exogenous; or chemically synthesized	Processed from long dsRNA; expressed from shRNA vector; or chemically synthesized	Expressed from shRNA vector	Endogenous; expressed from miRNA vector; or chemically synthesized
Risk of immunogenicity	High	Moderate	Low	Low
Level of complementary	Fully complementary to mRNA	Fully complementary to mRNA	Fully complementary to mRNA	Partial complementary to mRNA (3′UTR of mRNA)
mRNA target	Single target	Single target	Single target	Multiple targets

Notes: ds, double stranded; nt, nucleotide; UTR, untranslated region.

**Table 2:** Selected in vivo studies of inhaled RNAi therapy on the treatment of lung cancer in the past five years.

RNAi	Administration/Delivery System	Methods	Key Observations	Ref.	Year
Akt1 shRNA	Inhalation/SDA-PEI	400 µg of dual expression vector (shRNA and Pdcd4 cDNA) was administered to mouse with lung cancer twice a week for 4 weeks	Inhibition of Akt1 and overexpression of Pdcd4 synergistically suppressed lung tumorigenesis	[101]	2015
Bcl-2 siRNA	Intratracheal/PEI conjugated with DOX	20 μg siRNA and 10 μg DOX was administered to mouse on day 3, 10 and 17 after B16F10 cell implantation	Co-administration of siRNA and DOX enhanced antitumor effect compared with DOX or siRNA alone	[105]	2015
EGFP siRNA	Intratracheal/Chitosan (as dry powder)	30 μg siRNA (1.5 mg dry powder formulation) was administered to mouse with lung tumors stably expressing EGFP	EGFP expression in lung tumors consisting of EGFP cells was effectively silenced	[9]	2015
RPN2 PnkRNA	Intratracheal/Naked PnkRNA	15 µg of PnkRNA was administered to lung cancer xenograft mouse model as single dose or once a week for 3 weeks	Lung tumor growth was significantly inhibited without serious toxicity	[106]	2013
c-myc siRNA	Intratracheal/RGD gold NPs	0.3 or 1 pmol NPs were administered to mouse at week 12, 13, 14 and 15 after lung cancer induction	Tumor cell proliferation and tumor size were successful suppressed; the survival of animal was also prolonged	[107]	2013
NPT2b siRNA	Inhalation/GPT-SPE	500 µg of siRNA was administered to mouse with lung cancer twice a week for four weeks	Lung cancer growth, cancer cell proliferation and angiogenesis was suppressed, apoptosis was facilitated	[108]	2013
AIMP2-DX2 shRNA	Inhalation/Lentivirus	2 µg of shRNA was administered to AIMP2+/− mouse (susceptible to tumorigenesis) twice a week for four weeks	Expression of AIMPS-DX2 was suppressed	[103]	2013
Luciferase siRNA	Intratrachael/Chitosan (as dry powder)	30 µg siRNA (1.5 mg powder formulation) was administered to mouse with lung metastasis (tumor cells stably expressing luciferase gene) twice a day for two days	Significant inhibition of the increase in luminescence intensity in the lungs	[109]	2013
Bcl-2 and MRP1 siRNA	Inhalation/Lipid-based NPs	170 µg/kg of siRNA (1:1 Bcl-2:MRP1) to mouse orthotopic lung cancer model	Enhanced antitumor activity and reduced adverse effects (compared with intravenous treatment)	[110]	2013
Akt1 shRNA	Inhalation/GT–SPE	400 µg shRNA was administered to mouse with lung cancer twice a week for 4 weeks	Lung tumorigenesis was suppressed	[102]	2012

Notes: AIMP2-DX2, aminoacyl-tRNA synthetases (ARS)-interacting multifunctional protein 2 lacking exon 2; Akt1, RAC-alpha serine/threonine-protein kinase B; Bcl-2, B-cell lymphoma 2; DOX: doxorubicin; EGFP, enhanced green fluorescence protein; GPT-SPE: glycerol propoxylate triacrylate and spermine; GT–SPE, glycerol triacrylate–spermine; MRP1, multidrug resistance protein 1; NPT2b: sodium-dependent phosphate co-transporter 2b; Pdcd4: programmed cell death protein 4; PEI: polyethylenimine; RGD, Arginine-glycine-aspartic acid peptide; RPN2, ribophorin II; NPs, nanoparticles; SDA-PEI, sorbitol diacrylatepolyethylenimine; TAX, paclitaxel.

**Table 3:** Selected in vivo studies of using RNAi therapy on respiratory infections.

RNAi	Administration/Delivery System	Methods	Key Observations	Ref.	Year
**RSV**					
P protein siRNA	Intranasal/Naked siRNA or TransIT-TKO	5 nmol (~70 µg) of siRNA was administered to mouse at time of RSV and/or PIV infection	Animals were successfully protected from RSV and PIV infections specifically	[123]	2005
N protein siRNA	Intranasal/Naked siRNA	100 µg of siRNA was administered to mouse before infection (single dose) or after infection (multiple doses)	Significant reductions of viral load was achieved in both prophylactic and therapeutic regimens	[124]	2009
**Influenza A**					
NP, PB1 shRNA	Intranasal/Infasurf	60 µg of shRNA (expressing NP, PB, or NP + PB1) was administered to mouse 13 h before infection	Decreased lung viral titers in animals treated with shRNA expressing NP; the reduction was more significant with shRNA expressing both NP and PB1	[125]	2004
NP and PA siRNA	Intranasal and hydrodynamic/Oligofectamine	1.5 nmol (~20 µg) of siRNA was administered by hydrodynamic injection to mouse 16–24 h before infection, second dose was administered at the same time of infection by intranasal instillation	Treated animals were protected from lethal challenge with highly pathogenic viruses	[126]	2004
NP siRNA	Intravenous/PEI	120 µg of siRNA was administered to mouse 3 h prior to infection	A 94% drop of virus titers in the lungs of infected mice	[127]	2005
M2 and NP siRNA expression plasmids	Intravenous/PEI	100 µg of DNA was administered to mouse 15 h prior to infection	Significant reduction of lung virus titers; treated animals were partially protected from lethal virus challenge	[128]	2007
NP, PA and PB1 siRNA expression plasmids	Intravenous/adenovirus	100 µg of DNA was administered to mouse 18 h prior to infection	Virus production in mice was potently inhibited	[129]	2008
**Tuberculosis**					
XCL1 siRNA	Intratracheal/Naked siRNA	5, 10 or 15 µg of siRNA was administered to Mtb infected mouse	XCL1 expression in the lungs was significantly suppressed; decreased T lymphocytes, IFN-γ response and disorganized granulomatous lesions and high fibrosis	[130]	2009
TGF-β1 siRNA	Intratracheal/Naked siRNA	Three doses at 5 days intervals of 10 µg of siRNA per dose were administered to IL-10 knockout mouse starting on day 60 post-infection	Expression of antimicrobial mediators (NO and iNOS) was effectively increased along with the reduction of the bacterial load in the lungs of treated mice	[13]	2011

Notes: iNOS: nitric oxide synthase; Mtb: *Mycobacterium tuberculosis*; M2: influenza virus matrix protein; NO, nitric oxide; NP, nucleocapsid protein; P protein: phosphoprotein; PA: polymerase acidic protein; PB, polymerase basic protein; PEI, polyethyleneimine; PIV, parainfluenza virus; RSV, respiratory syncytial virus; TGF: transforming growth factor; XCL1, lymphotactin.

**Table 4:** Selected in vivo studies of using RNAi therapy on respiratory inflammatory diseases.

RNAi	Administration/Delivery System	Methods	Key Observations	Ref.	Year
**Asthma**					
SOCS siRNA	Intranasal/Naked siRNA	2 µM siRNA (~0.4 µg) was administered to OVA sensitized and challenged mouse (total 10 doses, every 3 days)	Decrease in lung eosinophilia, significant reduction of AHR, mucus secretion and collagen deposition in the airways	[12]	2014
IL-4 siRNA and P protein (anti-RSV) siRNA	Intranasal/Naked siRNA	100 µg of IL-4 siRNA (3 doses) and 70 µg of anti-RSV siRNA (single dose) were administered to OVA sensitized mouse with RSV-induced exacerbation	Eosinophilia in BALF, AHR and airway inflammation were significantly reduced	[165]	2014
STAT6 siRNA	Intratracheal and intranasal/Naked siRNA	2 mg/kg of siRNA was administered to OVA sensitized and challenged rat for 3 consecutive days	Allergen-induced lung inflammation was significantly reduced	[10]	2014
STAT6 siRNA	Intranasal/Naked siRNA	100 µg of siRNA (3 doses) was administered to OVA sensitized and challenged mouse	Expression of key cytokines (IL-4, IL-13) and allergen-induced inflammation in lung tissues were significantly reduced	[166]	2009
miR-1 mimic; mpl siRNA	Intranasal/Naked siRNA	50 µg of miRNA mimic or mpl siRNA was administered to OVA sensitized and challenged mouse	miR-1 mimic blocked the inflammatory responses and IL-13 expression; mpl knockdown reduced lung inflammation, Th2 cytokine secretion and mucus secretion	[167]	2013
Rip2 siRNA	Intratracheal/Naked siRNA	1 or 5 nmol of siRNA (~13 µg or 67 µg) was administered to OVA sensitized and challenged mouse for 3 consecutive days	OVA-induced cytokine release, inflammatory cell infiltration and mucus hypersecretion were inhibited	[168]	2013
Let-7 miRNA mimic	Intranasal/Naked miRNA	50–300 nM microRNA was administered to OVA sensitized and challenged mouse for 3 consecutive days	Expression of IL-13 was inhibited in the lungs ; suppression of airway inflammation and AHR	[169]	2011
GATA3 shRNA	Intratracheal/Lentivirus	2.2 × 10^6^ IFU of viral vectors were administered to OVA sensitized and challenged mouse	Expression of T_h_2 cytokines, antigen-induced airway inflammation and AHR were significantly inhibited	[170]	2008
CD86 siRNA	Intratrachael/Naked siRNA	12.5 µg of siRNA was administered to OVA sensitized and challenged mouse	OVA-induced airway eosinophilia, AHR and cytokines production was reduced	[171]	2014
Syk siRNA	Intranasal/Naked siRNA	10 µg of siRNA was administered to mouse for 3 consecutive days	The recruitment of inflammatory cells in the BALF of allergen sensitized mice was effectively inhibited	[172]	2013
c-kit siRNA	Intranasal/Naked siRNA	35 µg of siRNA was administered to OVA sensitized and challenged mouse for 3 consecutive days	Airway mucus secretion and eosinophil infiltration in BALF was effectively reduced	[173]	2014
Fluorescently labeled siRNA	Intratrachael/Tf-PEI	750 pmol of siRNA (~10 µg) was administered to OVA sensitized and challenged mouse for 4 consecutive days	Tf-PEI polyplexes selectively delivered siRNA to activated T cells	[174]	2016
**Acute Lung Injury**					
S1PLyase siRNA	Intratracheal/siRNA/HMGB1A/R3V6 ternary complex	300 pmol of siRNA (~4 µg) was administered to mouse model of ALI induced by LPS	Expression of IL-6 and TNF-α in BALF and lung tissues and the inflammatory response was significantly reduced	[11]	2014
TNF-α siRNA siRNA	Intratracheal/Naked siRNA; Intravenous/liposmes	50 µg TNF-α siRNA was administered to ALI mouse model	Systemic injection but not intratracheal delivery of TNF-α siRNA significantly reduced incidence of ALI	[175]	2012

Notes: AHR, airway hyperresponsiveness; ALI, Acute lung injury; BALF, bronchoalveolar lavage fluid; CD86, cluster of differentiation 86; C-kit, a stem cell factor receptor; DCs, dendritic cells; HMGB1A, high mobility group box-1 A peptide; IFU, infectious unit; LPS, lipopolysaccharide; Mpl, myeloproliferative leukemia virus oncogene; OVA, ovalbumin; R3V6, an arginine-rich peptide; Rip2, receptor-interacting protein 2; RSV, respiratory syncytial virus; S1Plyase, sphingosine-1-phosphate lyase, SOCS, Suppressors of cytokine signaling protein 3; STAT6, signal transducer and activator of transcription factor 6; Syk, spleen tyrosine kinase; Tf-PEI, transferrin polyethylenimine; T_h_2, T helper 2 cells; TNF-α, tumor necrosis factor-α; VEGFR, Vascular endothelial growth factor.

**Table 5:** Selected in vivo studies of using RNAi therapy on pulmonary fibrosis.

RNAi	Administration/Delivery System	Methods	Key Observations	Ref.	Year
TGF-β1, CTGF, PDGF, CCL2, and Tissue factor siRNA	Intratracheal/Naked siRNA	0.05–5 mg/kg of siRNA (~1–100 µg) was administered to mouse models of pulmonary fibrosis (total 3 doses)	Pulmonary fibrosis was strongly inhibited by TGF-β1 and CCL2 siRNAs, weakly inhibited by CTGF, PDGF and tissue factor siRNAs	[224]	2012
CTGF siRNA	Intratracheal/PEGylated PDMAEMA	10–50 µg of siRNA was administered to bleomycin-induced rat model of pulmonary fibrosis	Collagen deposition and inflammatory cytokines production was significantly reduced	[225]	2013
miR-326 mimic and TGF-β1 siRNA	Intranasal/Naked siRNA	90 or 120 µg miRNA mimic, or 120 µg siRNA was administered to bleomycin-induced mouse model of pulmonary fibrosis (total 4 doses)	Both treatments inhibited the expression of TGF-β1 and attenuated the fibrotic response	[92]	2014
AR and CTGF siRNA	Intratracheal or intravenous/SAMiRNA nanoparticles	1–5 mg/kg SAMiRNA was administered to bleomycin or TGF-β transgenic mouse model of pulmonary fibrosis	Collagen accumulation in the lung of animal was effectively inhibited	[8]	2016

Notes: AR, amphiregulin; CCL2, chemokine (C-C motif) ligand 2/monocyte chemoattractant protein-1; CTGF, connective tissue growth factor; PDGF, platelet-derived growth factor; PDMAEMA, poly(dimethylamino)ethylmethacrylate; PMAPEG: poly(methylether-methacrylate-ethyleneglycol); SAMiRNA, self-assembled micelle interfering RNA; TGF-β, transforming growth factor β.

**Table 6:** Summary of clinical trials of RNAi therapeutics in respiratory diseases.

RNAi	Condition	Delivery	Status	Company/Sponsor	Ref/Trial ID	Year
N protein siRNA (ALN-RSV01)	RSV infection	Intranasal, naked siRNA	Phase I & II Completed	Alnylam Pharmaceuticals	[139,140,230]	2012
N protein siRNA (ALN-RSV01)	RSV infection in lung transplant patient	Inhalation, naked siRNA	Phase II completed	Alnylam Pharmaceuticals	[231]	2011
Syk siRNA (Excellair)	Asthma	Inhalation, naked siRNA	Phase I completed	ZaBeCor Pharmaceuticals	[199,200]	2010
miR-34 (MRX34)	Solid tumor including NSCLC	Intravenous, liposomes	Phase I ongoing	Mirna Therapeutics	NCT01829971	2013
miR-16 (TargomiRs)	Recurrent MPM and NSCLC	Intravenous, nanoparticles	Phase I ongoing	University of Sydney	NCT02369198	2015

Notes: MPM, malignant pleural mesothelioma; NSCLC, non-small cell lung cancer; RSV, respiratory syncytial virus; Syk, spleen tyrosine kinase.
